# Influence of medium chain fatty acids on selected microbes and on *in vitro* ruminal fermentation of air-exposed corn silage

**DOI:** 10.3389/fvets.2024.1416695

**Published:** 2024-09-11

**Authors:** Jaime Salinas-Chavira, Claudio Arzola-Alvarez, Michael E. Hume, Mozart Fonseca, Oscar Ruiz-Barrera, Yamicela Castillo-Castillo, Marina Ontiveros-Magadan, Barbara Jones, Tawni L. Crippen, Toni L. Poole, Aracely Zuñiga-Serrano, Robin C. Anderson

**Affiliations:** ^1^College of Veterinary Medicine and Animal Science, Autonomous University of Tamaulipas, Ciudad Victoria, Tamaulipas, Mexico; ^2^College of Animal Science and Ecology, Autonomous University of Chihuahua, Chihuahua, Mexico; ^3^United States Department of Agriculture, Agricultural Research Service, Food and Feed Safety Research Unit, College Station, TX, United States; ^4^Department of Agriculture, Nutrition and Veterinary Sciences, University of Nevada, Reno, NV, United States; ^5^Departamento de Ciencias Veterinarias, Autonomous University of Ciudad Juarez, Ciudad Juarez, Chihuahua, Mexico; ^6^Department of Animal Science and Veterinary Technology, Tarleton State University, Stephenville, TX, United States

**Keywords:** fatty acids, aerobic phase, corn silage, microbes, rumen

## Abstract

Several medium chain fatty acids and different chemical forms of these acids were evaluated *in vitro* as treatments of aerobically-exposed corn silage against spoilage and pathogenic microbes and for effects on rumen fermentation. Treatments were control (no additive), myristate (MY), laurate (LA), monolaurin (MLA), methyl ester laurate (MELA), a blend of mono-, di- and triglycerides of laurate (BLA), and monocaprylate (MC). After 24 h of aerobic incubation (37°C), yeast and mold growth were not influenced (*P* > 0.05) by treatments, while the net growth of lactic acid bacteria was decreased, albeit slightly, compared to that by untreated controls (*P* < 0.01) by all treatments of the air-exposed corn silage. Compared with controls, wild-type enterococci were decreased (*P* < 0.01) in MLA, MELA, and BLA. *Staphylococcus aureus* was reduced (*P* < 0.01) with MLA, MELA, BLA, and MC. Total aerobes showed reductions (*P* < 0.01) with MLA, BLA, and MC. *Listeria monocytogenes* numbers were reduced (*P* < 0.01) with MELA. Anaerobic incubation (24 h; 39°C) of ruminal fluid (10 mL) with 0.2 g air-exposed and MCFA-treated corn silage revealed higher hydrogen accumulations (*P* < 0.01) with MLA and MC over the control treatment. Methane was decreased (*P* < 0.01) solely by MLA. There was an increase (*P* < 0.01) of acetate with MELA and MC; of propionate with MELA or by BLA; and of butyrate with MLA, MELA, BLA, and MC. Total VFA, hexose fermented, and ammonia were increased (*P* < 0.01) with MELA, BLA, and MC. The acetate:propionate ratio was increased (*P* < 0.01) with MC. The results showed that treatment of air-exposed corn silage with esterified MCFA had no effect on yeasts and molds but prevented propagation or reduced populations of some unwanted and potentially desirable bacteria. Modest methane reduction was seen during *in vitro* incubation of rumen fluid suspensions with MLA-treated silage and ammonia accumulations were increased in esterified MCFA-treated silage. Little, if any, other detrimental effects on beneficial ruminal fermentation characteristics were observed.

## 1 Introduction

The opening of the anaerobic seal of silages during feed-out starts irreversible aerobic changes that alter the nutritional and microbiological quality of the feedstuff, and this process continues in the feed bunk. Yeasts and molds are the main sources of aerobic spoilage microorganisms, with some bacterial sources serving as secondary contributors (1). Microbial safety of the silage could be compromised during the aerobic phase, with the growth of pathogenic bacteria including *Staphylococcus aureus, Listeria monocytogenes*, Enterobacteriaceae (*Escherichia coli* and *Salmonella* spp.) as well as *Clostridium botulinum* (2, 3). In addition, some molds may produce mycotoxins with the potential to disturb animal production and health (1).

The medium chain fatty acids (MCFA) octanoic (C8, caprylate), decanoic (C10, caprate) and dodecanoic (C12, laurate), and their monoglycerides, have the capacity to reduce numbers of Gram-positive bacteria (4, 5). Moreover, caprylate inhibited the growth of enteropathogenic bacteria (6–10). During silage preparation, MCFA also have shown influences on fermentation. McDonald and Henderson (11) observed reduced fermentation with hexanoic acid (C6, caproates), while Abel et al. (12) found reduced lactate content in grass-silage treated with caprylate. Additionally, the proliferation of yeasts and molds have been reduced in silage treated with propionic acid (C3, propionate) (13).

MCFA consistently have reduced methane production during ruminal fermentation (14–16). However, when MCFA have been added at the time of ensiling they have not reduced methane production following ingestion and subsequent fermentation in the rumen (12). There is limited information regarding the effects of MCFA when applied in silages at the time of opening as a possible treatment to inhibit or delay silage deterioration or promote the control of pathogenic bacteria during the aerobic phase. Also, there are limited reports regarding the residual effects of MCFA applied at the silage opening on ruminal fermentation characteristics. Ontiveros-Magadan et al. (17), with air-exposed silage treated with laurate (LA) or monolaurin (MLA), observed reduced numbers of Gram-positive bacteria, yeasts, and molds, although the numbers of lactic acid bacteria (LAB) were reduced after a few hours of incubation; in addition, methane production also was reduced during *in vitro* ruminal incubations. In a similar study, Arzola-Alvarez et al. (18) used MCFA (C6; C8; C10; a C8:10 mixture; and a C6:12 mixture) and found reductions of enterococci and LAB, although *Staphylococcus aureus* and *Listeria monocytogenes* were unaffected by treatment, and yeast and mold were solely reduced with the C8:10 mixture. Those authors reported small changes in ruminal fermentation characteristics.

As stated, information on the treatment of air exposed corn silage with MCFA is limited and inconsistent regarding microbial changes during the aerobic phase, and on possible residual effects on rumen fermentation characteristics. In addition, to the best of our knowledge, there is no information on the application in silage of methyl ester laurate, blends of mono-, di-, and triglycerides of laurate, or monocaprylate. Considering the importance of maintaining nutritional quality and reducing the proliferation of unwanted microorganisms during the aerobic exposition of silage, the objective of the present experiment was to test our hypothesis that treatment of aerobically-exposed corn silage with MCFA and MCFA esters will prevent propagation of select spoilage and pathogenic microbes without having adverse carry over effects on rumen fermentation when incubated *in vitro* under ruminal habitat simulating conditions.

## 2 Materials and methods

### 2.1 Sources of microbes

*Listeria monocytogenes* strain Scott A (serotype 4b) was provided by Dr. J. F. Frank, University of Georgia, Athens, GA, USA. A multi-drug resistant/methicillin-resistant (MDR) *Staphylococcus aureus* strain ATCC 49521, demonstrated earlier to be resistant to erythromycin, linezolid, penicillin, quinupristin/dalfopristin, and vancomycin (19), was provided by the late Dr. Max Paape. Stock cultures grown overnight at 37°C in aerobic tryptic soy broth (Becton Dickinson and Company, Sparks, MD, USA) were used as inocula for incubations with air-exposed silage. Corn silage (38 ± 5.3% dry matter and pH 4.73) was collected at the Southwest Regional Dairy Center at Tarleton State University (Stephenville, TX, USA) and stored at room temperature until use ~24 h later.

Ruminal fluid used for *in vitro* tests of potential carry-over effects of MCFA in treated silage was collected the morning of the experiment from a cannulated Jersey cow grazing Bermudagrass pasture with *ad libitum* access to alfalfa hay at the USDA Southern Plains Agricultural Research Center, College Station, TX. All procedures for the care and use of this cow were approved by the Southern Plains Agricultural Research Center's Institutional Animal Care and Use Committee.

### 2.2 Tests with mixed populations of silage and rumen bacteria

Four-gram portions air-exposed silage were placed into separate triplicate sets (*n* = 3/treatment) 50-mL conical centrifuge tubes that had had been loaded previously without or with 30 mg of the test MCFA. For this study, the MCFA used were myristate (MY; tetradecanoic acid), laurate (LA; dodecanoic acid), monolaurin (MLA; glycerol monolaurate), methyl ester laurate (MELA), a blend of mono-, di-, and triglycerides of laurate (BLA), or monocaprylate (MC; glycerol monocaprylate). All MCFA used in this study were supplied by Berg + Schmidt GmbH & Co. KG (Hamburg, Germany). Sterile distilled water (10 mL) was added to each of the tubes prepared above and each tube was inoculated with 10 μL each of *L. monocytogenes* Scott A and *S. aureus* ATCC 49521. Each inoculum had been grown aerobically for 20 h at 37°C in tryptic soy broth (Becton Dickinson and Company) prior to inoculation. Thereafter, the air-exposed silage preparations were capped and incubated at 30°C while exposed to air. Liquid samples collected upon initiation and completion of the 24 h incubation period were then serially diluted (10-fold) in 0.1 M sodium phosphate buffer (pH 6.5) and then plated onto solidified selective and differential medium for viable cell count of select microbial populations. The experimentally inoculated *S. aureus* and *L. monocytogenes* strains were respectively enumerated using BBL™ Mannitol Salt (Becton Dickinson and Company) and *Listeria* Selective Agar supplemented with Oxoid™ *Listeria* Selective Supplement (Oxford LTD, Basingstoke, Hampshire, England). Wildtype lactic acid bacteria (LAB) and enterococci were enumerated using Rogosa SL and m *Enterococcus* agars, respectively (each sourced from Becton Dickinson and Company). Respective counts of total aerobes and yeasts and molds were enumerated using 3M™ Petrifilm™ Aerobic Count Plates and 3M™ Petrifilm™ Yeast and Mold Count Plates (3M Petrifilm, St. Paul, MN, USA). Inoculated plates and petrifilm were used according to manufacturer's instructions.

To examine potential carryover effects of the MCFA in treated air-exposed silage on rumen fermentation, the air-exposed corn silage that had been treated individually without or with MY, LA, MLA, MELA, BLA, or MC were incubated *in vitro* under ruminal habitat simulating conditions using ruminal fluid collected freshly at 10:00 the morning of the study from a ruminally-cannulated Jersey steer grazing Bermudagrass pasture. The ruminal fluid, containing viable populations of rumen microbes, was obtained by squeezing rumen contents collected via the cannula through a nylon paint strainer (20) into a 250-mL thermos which was immediately capped upon filling and returned to the laboratory within 30 min of collection. The collected rumen fluid was distributed in 10 mL volumes under a continuous flow of carbon dioxide into 18 x 150 mm crimp culture top tubes (3 replicate tubes/treatment) that had been preloaded without or with 0.2 gram aerobically exposed silage that had been treated individually without or with MY, LA, MLA, MELA, BLA, or MC. The tubes were then capped, sealed and incubated for 24 h at 39°C. Culture fluids and headspaces gases collected at the beginning and end of incubation were subjected to gas chromatography for determination of volatile fatty acids (VFA) (21) and for hydrogen and methane content (22). Headspace gas volumes at the end of incubation were measured by volume displacement using a glass syringe. Concentrations of ammonia were measured colorimetrically (23). Stoichiometric estimates of the amounts of hexose fermented were calculated as ($\frac{1}{2}$ acetate + $\frac{1}{2}$ propionate + butyrate + valerate) and fermentation efficiency was calculated as (0.62 acetate + 1.09 propionate + 0.78 butyrate)/(acetate + propionate + butyrate), Chalupa (24). Bleach, nitroferricyanide, phenol, and sodium hydroxide used in the ammonia assay were obtained from CloroxTM (Oakland, CA, USA), Fisher (Fair Lawn, NJ, USA), Sigma-Aldrich (St. Louis, MO, USA), and Argos Organics (Geel, Belgium). Mono and disodium phosphate used to prepare 0.1 M phosphate buffer were purchased from Sigma.

### 2.3 Statistical analysis

Control and treated cultures were each incubated in triplicate, with each replicate serving as an independent experimental unit. Net changes in viable bacterial counts, expressed as log_10_ colony forming units (CFU)/g of silage, net accumulations of ruminal fermentation products, and stoichiometric estimates of amounts of hexose fermented were determined as the difference between measurements made at the beginning and end of incubation, and were analyzed for potential treatment effects using a completely randomized analysis of variance with a two-sided Dunnett's multiple comparison to controls (Statistix10 Analytical Software, Tallahassee, FL, USA).

## 3 Results

### 3.1 Selected microbial concentrations

The effects of the different MCFA on the net change, representing the difference between initial and final concentrations, in microbial concentrations during the incubation are shown in Table 1. In the case of *S. aureus*, the 1.51–1.90 log-fold decrease observed in aerobically-exposed silage treated with MC, BLA, MLA, and MELA differed (*P* < 0.05) from the 0.14 log_10_ CFU/g increase observed with the untreated controls. The net change in *S. aureus* concentrations observed with MY and LA did not differ (*P* > 0.05) from that observed with the controls. Similarly, total aerobe concentrations showed reductions from their initial levels when the air-exposed silage was treated with MLA, BLA, or MC but the magnitude of the decrease was much more modest that that observed with *S. aureus*. Nevertheless, the net change in total aerobes were significantly different with the MLA-, BLA-, and MC-treated silage (*P* < 0.01) than the net change observed with the untreated controls (Table 1). However, concentrations of total aerobes were similar (*P* > 0.05) between the control group and those treated with LA, MY, or MELA.

**Table 1:** Influence of medium chain fatty acids on microbial populations.

	**Medium chain fatty acids (treatments)**							**SEM**	***P*-value**
**Microbes**	**Control**	**MY (C14)**	**LA (C12)**	**MLA**	**MELA**	**BLA**	**MC**		
**Net change in log**_10_ **CFU/g of silage**^a^									
*Staphylococcus aureus*	0.14	−0.52	−0.80	−1.80^*^	−1.90^*^	−1.59^*^	−1.51^*^	0.33	<0.01
Total aerobes	0.80	0.70	0.18	−0.60^*^	−0.30	−0.64^*^	−0.63^*^	0.27	<0.01
Lactic acid bacteria	0.38	0.35^*^	0.24^*^	0.16^*^	0.21^*^	0.17^*^	0.17^*^	0.03	<0.01
Enterococci	−1.01	−1.68	−1.45	−2.27^*^	−2.61^*^	−2.94^*^	−1.94	0.25	<0.01
Yeast and molds	0.86	0.62	0.48	0.29	0.13	0.52	0.17	0.20	0.18
*Listeria monocytogenes*	−0.66	−0.17	−0.76	−1.36	−1.98^*^	−1.26	−1.39	0.32	0.03

Control (no additive); MY, myristate; LA, laurate; MLA, monolaurin; MELA, methyl ester laurate; BLA, blend of mono-, di-, and triglycerides of laurate; MC, monocaprylate.

^a^Expressed as the difference between times 0- and 24-h log_10_ CFU/g.

^*^Means (n = 3) in each row and with asterisks differ significantly from controls (P < 0.05).

Populations of LAB increased slightly, by 0.17–0.38 log_10_ CFU/g in all treated silages as well as the untreated silage, but the increase was less than in the untreated control for all treated silages (*P* < 0.01), including those with the non-esterified free forms or the esterified forms of the MCFA (Table 1). Populations of enterococci were decreased from their initial levels by 10- to 100-fold in the untreated as well as all treated silages, but the net change from initial levels in MLA-, MELA-, and BLA-treated silages was significantly different (*P* < 0.01) from the net change in the controls. The net change in concentrations of yeast and molds, when compared to the net change observed in untreated controls, did not differ (*P* > 0.05), with populations increasing more variably from 0.13 to 1.39 log_10_ CFU/g (Table 1). The experimentally inoculated *L. monocytogenes* Scott A population also responded variably to the MCFA treatments in the air-exposed corn silage, with populations being decreased from their initial concentrations in all treated and untreated silages (Table 1). However, the 1.98 log_10_ CFU/g decrease observed in the MELA-treated silage was the only treated-silage that differed significantly (*P* < 0.05) than the net change of −0.66 log_10_ CFU/g observed with the untreated controls (Table 1).

### 3.2 *In vitro* rumen fermentation

A summary of the effects of MCFA on *in vitro* ruminal fermentation characteristics is shown in Table 2. Hydrogen accumulations in the *in vitro* rumen fluid suspension incubated with silage that had been treated with MLA or MC was increased (*P* < 0.01) compared accumulations observed in the control rumen fluid suspensions incubated *in vitro* with silage that had not been treated with any of the MCFA preparations (Table 2). Methane was decreased (*P* < 0.01) 23% in the rumen fluid suspensions incubated *in vitro* with the addition of MLA when compared to that produced by the control rumen suspensions incubated with non-treated silage (Table 2). Accumulations of acetate were increased (*P* < 0.01) more than 2-fold in the rumen fluid suspensions incubated *in vitro* with silage treated with MELA or MC when compared to the amount produced by the control rumen fluid suspensions incubated with the untreated silage (Table 2). Propionate accumulations were increased (*P* < 0.01) more than 1.8-fold in the rumen suspensions incubated with MELA or BLA when compared accumulations in the control rumen fluid suspensions (Table 2). Accumulations of isobutyrate and isovalerate in the rumen fluid suspensions incubated with the treated silages did not differ (*P* > 0.05) compared to accumulations observed in the control rumen fluid incubations (Table 2). However, butyrate accumulations in the rumen fluid suspensions incubated with MLA, MELA, BLA or MC were increased (*P* < 0.01) by 1.7- to 2-fold when compared to control incubations (Table 2). The accumulation of valerate in the rumen fluid suspensions incubated with MLA was increased (*P* < 0.01) 3.6-fold when compared to the control rumen fluid incubations. Owing to the increased accumulations of individual fatty acids observed above, accumulations of total VFA and stoichiometric estimates of hexose fermented in the *in vitro* rumen fluid incubations were increased (*P* < 0.01) by 1.8 to 2-fold due to silage treated with MELA, BLA, or MC (Table 2). The acetate:propionate ratio was increased (*P* < 0.05) 2-fold in the rumen fluid suspensions incubated with MC-treated silage when compared to the control rumen fluid incubations which consequently contributed to a 6.4% decrease (*P* < 0.05) in the estimated fermentation efficiency of the MC-treated silage incubations (Table 2). Ammonia accumulations were increased (*P* < 0.01) by 13 to nearly 18-fold in rumen fluid suspensions incubated with MELA, BLA, or MC when compared to the control rumen fluid incubations (Table 2).

**Table 2:** Influence of medium chain fatty acids on *in vitro* ruminal fermentation characteristics.

**Culture component**	**Medium chain fatty acids (treatments)**							**SEM**	***P*-value**
	**Control**	**MY (C14)**	**LA (C12)**	**MLA**	**MELA**	**BLA**	**MC**		
Hydrogen^a^, μmol/mL	0.10	0.04	0.04	0.72^*^	0.16	0.24	1.49^*^	0.08	<0.01
Methane^a^, μmol/mL	10.90	9.84	9.68	8.37^*^	11.38	10.55	10.56	0.50	0.02
Acetate, μmol/mL	12.13	17.02	10.71	16.94	26.81^*^	21.91	24.76^*^	2.80	<0.01
Propionate, μmol/mL	6.01	7.69	5.29	9.24	11.25^*^	10.88^*^	6.08	0.99	<0.01
Isobutyrate, μmol/mL	0.18	0.24	0.13	0.20	0.29	0.30	0.25	0.41	0.11
Butyrate, μmol/mL	7.34	9.13	7.11	12.68^*^	13.85^*^	13.38^*^	14.43^*^	1.03	<0.01
Isovalerate, μmol/mL	0.53	0.71	0.52	0.61	0.91	0.86	0.66	0.10	0.06
Valerate, μmol/mL	0.49	0.61	0.46	1.78^*^	0.97	1.17	1.06	0.19	<0.01
Total VFA^b^, μmol/mL	16.91	22.10	15.57	27.55	33.85^*^	30.94^*^	30.90^*^	2.95	<0.01
Fermentation efficiency, %	78.45	77.02	77.92	78.54	76.47	77.72	73.39^*^	0.55	<0.01
Hexose fermented, μmol/mL	26.69	35.41	24.21	41.45	54.09^*^	48.49^*^	47.23^*^	4.95	<0.01
Acetate:Propionate ratio	1.85	2.21	1.96	1.81	2.38	2.01	4.13^*^	0.18	<0.01
Ammonia, μmol/mL	0.07	0.03	−0.05	0.34	0.93^*^	1.16^*^	1.23^*^	0.14	<0.01

Control (no additive); MY, myristate; LA, laurate; MLA, monolaurin; MELA, methyl ester laurate; BLA, blend of mono, di and triglycerides of laurate; MC, monocaprylate.

^a^Headspace gases.

^b^VFA, volatile fatty acids.

^*^Means (n = 3) in each row and with asterisks differ significantly from controls (P < 0.05).

## 4 Discussion

### 4.1 Selected microbial concentrations

Laureate or myristate did not have an influence on *S. aureus* growth; however, numbers of this bacterium were reduced 10- to 100-fold when silages were treated with the monoglycerides of caprylic or lauric acids, as well as MELA. In agreement, Arzola-Alvarez et al. (18) did not report changes in *S. aureus* growth in air-exposed corn silage treated with MCFA. However, these results contrast with those of Ontiveros-Magadan et al. (17) in a similar study with air-exposed corn silage. They observed reductions in *S. aureus* populations in silages treated with LA or with MLA (a glyceride of laureate). Probably, these different results could be attributable to the different doses of MCFA used. In the current study and in the study of Arzola-Alvarez et al. (18) 0.3 mg/kg of MCFA was used, while Ontiveros-Magadan et al. (17) used 0.5 mg/kg of MCFA. In addition to the dose, a theme that needs further research relates to the efficacy of the chemical forms of MCFA to inhibit or reduce the *S. aureus* growth in air-exposed corn silage, considering that in the present study, the glycerides of MCFA were more effective than the free fatty acids (laureate or myristate). For instance, Kelsey et al. (25), in pure cultures of *S. aureus*, reported similar decreased growth with treatments of the MCFA laurate, caprate, myristate, and glycerol monolaurate.

Laureate, myristate, or methyl ester lauric acid had little, if any, negative influence on total aerobes growth; however, the monoglycerides of caprylic and lauric acids reduced total aerobe populations but the decrease was <10-fold. As reviewed others (26, 27), it is not unexpected that the monoglycerides of medium chain fatty acids, possessing surfactant and easier micelle-forming characteristics, may exhibit more potency than the free fatty acids against certain but not necessarily all microbes. Arzola-Alvarez et al. (18) did not report changes in total aerobe growth in air-exposed corn silage treated with MCFA in free form. However, these results contrast with those of Ontiveros-Magadan et al. (17) who found total aerobe reductions when silages were treated with LA or with MLA. As previously mentioned, the doses used in the different studies could be related to the differences in the results.

All of the fatty acids moderately reduced the net growth of LAB, when compared to controls indicating a modest bacteriostatic, rather than a bactericidal effect, the latter which would be manifested as a decrease in concentrations from their initial level. According to Ellis et al. (28) lactic acid bacteria generally consist of multiple different genera of Gram-positive bacteria such as certain *Bacillus* species as well as species belonging to *Enterococcus, Lactobacillus, Lactococcus, Pediococcus*, and *Weissella* (27). Accordingly, the decrease in LAB in the treated silage may be due, at least partially, to the observed decrease in enterococci discussed below. The current results are consistent with those of Arzola-Alvarez et al. (18) who observed LAB reductions with treatments of MCFA in air-exposed corn silage. In pure cultures of microorganisms related to silage, Woolford (29) reported that the increase in chain-size of fatty acids had greater antimicrobial effect. In addition, they reported that caprylic (octanoic), capric (decanoic), and lauric (dodecanoic) acids were effective against LAB, yeast, molds, and Gram-positive bacteria, while Gram-negative bacteria were less sensitive than Gram-positive bacteria to the tested fatty acids. Gram-positive bacteria, which lack an outer membrane layer, are generally recognized as being more susceptible to cell wall damage caused by the MCFA or their esters than are Gram-negative bacteria, presumably due to the limited ability of the compounds to pass through the outer membrane layer of Gram-negative bacteria (26). In different results, Ontiveros-Magadan et al. (17) did not find changes in the growth of LAB at 24 h of incubation in air-exposed corn silage treated with LA or MLA. LAB are wanted bacteria which maintain the acid conditions in silages; however, when the silage is air-exposed, the LAB exhibit reduced growth, and, in the study of Ontiveros-Magadan et al. (17), LAB were absent in the control group and had only small changes in the air-exposed silages treated with LA or MLA.

Laureate and myristate had no influence on enterococci growth, however methyl ester and the glycerides of lauric acid did reduce their numbers. Enterococci growth has been shown to be reduced in air-exposed corn silage treated with MCFA (18). Additionally, Bunkova et al. (5) observed *in vitro* inhibitory growth of Gram-positive bacteria (*Bacillus cereus, B. subtilis, E. faecalis, Micrococcus luteus*, and *S. aureus*) treated with monoglycerides containing MCFA including C8, C10, C12, and other fatty acids. They also reported that the Gram-negative bacteria tested (*Citrobacter freundii, E. coli, Proteus mirabilis, Pseudomonas aeruginosa*, and *Salmonella enterica*) were more resistant to the same monoglycerides.

In the current study, treatments did not influence the growth of yeast and molds in air-exposed corn silage. Growth inhibition of these microorganisms is a desirable target due to the spoilage effect on silages during the aerobic phase after opening the silo. There is no consistent effect of MCFA on the growth of yeast and molds. Woolford (29), using pure cultures, reported reductions in numbers of yeasts and molds associated with silages when treated with C10 and C12 fatty acids. Arzola-Alvarez et al. (18) found reductions in the numbers of yeast and molds by a blend of C8:C10 fatty acid treatment in air-exposed silage; however, there were no reductions when the same fatty acids were used separately. Ontiveros-Magadan et al. (17), using air-exposed corn silage treated with LA (C12), found yeast and molds reductions; and the same authors noted that from a practical perspective, those reductions would be modest and inconsequential.

*Listeria monocytogenes* populations were only decreased by the MELA. In agreement, Arzola-Alvarez et al. (18) did not report changes in *L. monocytogenes* growth in air-exposed corn silage treated with MCFA in free forms. In pure cultures, *L. monocytogenes* had reduced populations when treated with MLA (30, 31). Ontiveros-Magadan et al. (17), using air-exposed corn silage, observed reductions in *L. monocytogenes* populations treated with LA or with MLA (a glyceride of laureate), although the reductions were more evident with LA than with MLA. As previously stated, concentrations of MCFA could account for these different responses on *L. monocytogenes* growth.

### 4.2 *In vitro* rumen fermentation

Hydrogen production was increased by monolaurin and mono-caprylic glycerides, while LA and MY did not influence hydrogen production. In agreement, previous research with air-exposed corn silage showed MCFA had minimal influence on rumen fermentation (17, 18). In the present study, only MC had hydrogen accumulation that was associated with negative effects on fermentation efficiency, the acetate:propionate ratio, or ammonia production.

In the current study, the MCFA had minimal influence on methane production, being only reduced by monolaurin. In agreement, Arzola-Alvarez et al. (18) using air-exposed corn silage reported minimal influence of MCFA on rumen methane production. In contrast, Ontiveros-Magadan et al. (17) found significant methane reductions with LA and MLA in air-exposed corn silage; as stated, this difference cold be due to the greater dose of MCFA in the study of Ontiveros-Magadan et al. (17). Lauric acid showed reduced methane at LA high concentrations (14). Goel et al. (32) observed diminished rumen methane production *in vitro* with capric acid at 400 and 600 mg/l, but no reductions were observed at 200 mg/l.

Methyl ester lauric acid and mono-caprylic glycerides increased acetate production. This result agreed with those of Arzola-Alvarez et al. (18), using air-exposed corn silage, who reported minimal influence of MCFA on rumen acetate production. Jordan et al. (33) also found minimal effect of coconut oil (rich in MCFA) on proportions of acetate, propionate, and butyrate, with total mixed rations for beef cattle. Abel at al. (12) observed greater acetate and methane content in silages treated with caprylic acid (C8) than in silage treated with capric (C10).

Methyl ester lauric acid and the lauric acid blend increased propionate. This result agreed with those of Arzola-Alvarez et al. (18) using air-exposed corn silage, and who reported minimal influence of MCFA in free forms on rumen propionate production. Jordan et al. (33) also found minimal effect of coconut oil (rich in MCFA) on proportions of acetate, propionate, and butyrate, with total mixed rations for beef cattle. Abel et al. (12) did not observe greater propionate content in silages treated with caprylic acid (C8) than in silage treated with capric (C10).

Butyrate content was increased by MLA, MC, MELA, and by the BLA. These results agree with those of Arzola-Alvarez et al. (18), using air-exposed corn silage, who reported minimal influence of MCFA in free forms on rumen butyrate production. Jordan et al. (33) also found minimal effect of coconut oil (rich in MCFA) on proportions of acetate, propionate, and butyrate with total mixed rations for beef cattle. Abel at al. (12) did not observe greater butyrate content in silages treated with caprylic acid (C8) than in silage treated with capric (C10).

Isobutyrate and isovalerate content were not influenced by treatments, and valerate content was increased only by MLA. These results agreed with those of Arzola-Alvarez et al. (18) using air-exposed corn silage. Abel at al. (12) did not observe greater isobutyrate levels in silages treated with caprylic acid (C8) than in silage treated with capric (C10).

Total VFA, hexose fermented, and ammonia were increased by MELA, BLA, and by MC. These results agree with those of Arzola-Alvarez et al. (18) working with air-exposed corn silage and reported minimal influence of MCFA on the same variables of rumen fermentations. Different results were reported by Jordan et al. (33) who saw reductions of total VFA content in mixed rations for beef cattle. Abel at al. (12) observed greater ammonia content reductions in silages treated with caprylic acid (C8) than in silages treated with capric (C10).

Mono-caprylic glyceride reduced fermentation efficiency and increased the acetate:propionate ratio. These are non-wanted fermentation characteristics in the rumen and could be related to hydrogen accumulations seen with this treatment. In the current study, fermentation efficiency was not influenced by LA or MY; however, in contrast, Arzola-Alvarez et al. (18) found reduced fermentation efficiency with MCFA. More research is warranted on this theme, particularly on the residual effect in treated silages of the different MCFA on ruminal fermentation characteristics.

Ruminal ammonia production was not modified by LA, MY, or by MLA. Conversely, ammonia content was greater, albeit not necessarily significantly, in the rumen fluid suspensions incubated with silage treated with the esterified MCFA than with the free fatty acids. Considering that esterified MCFA treatments of air exposed silage resulted in appreciable decreases in staphylococci, enterococci and lactate bacterial concentrations, it is reasonable to suspect these bacterial populations may be correlated with appreciable ammonia uptake within with rumen microbiota. The lack in response in air-exposed corn silage to MCFA on ruminal ammonia production agreed with results of Arzola-Alvarez et al. (18). However, consistently, ruminal ammonia production has been reduced with LA supplementation, although amounts of lauric acid administered to the cattle in those studies (240 g/cow per day) would likely have exceeded the amount, 0.15 mg/mL of rumen fluid basis, included in our *in vitro* incubations (34, 35). Assuming a 60-liter rumen volume of the 660–680 kg cows, the maximum rumen concentration would have been ~4 mg of laurate/mL. Ruminal ammonia reduction is a desirable effect of LA because of reduced microbial proteolysis and reduced microbial populations, with the net effect of nitrogen improvement by the ruminant animal. Indications from our study are that a higher dose fatty acids may cause a reduction in ammonia content in the rumen fermentation.

## 5 Conclusion

Results from the present study provide support to our hypothesis that treatment of aerobically-exposed corn silage with MCFA preparations of MLA, MELA, BLA or MC not only prevented the propagation of experimentally-inoculated *S. aureus* but achieved near 2 log_10_-fold reductions in colony counts of this mastitis-causing pathogen. However, these treatments also caused significant, albeit slight (<1 log_10_-fold), decreases in potentially beneficial populations of indigenous lactic acid bacterial populations as well as near 2 log_10_-fold reductions in enterococcal populations. Effects of these MCFA preparations as well as the free forms of MY or LA on total aerobes, yeast and molds and on experimentally-inoculated *L. monocytogenes* were variable. Tests for potential adverse carry over effects of the MCFA treated silage on rumen fermentation during *in vitro* incubation under ruminal habitat simulating conditions revealed little, if any, detrimental effects on beneficial ruminal fermentation characteristics with the exception of significant increases in ammonia accumulations in rumen fluid suspensions incubated with MELA, BLA, and MC which implicates potential an effect on ammonia assimilation within the mixed population of rumen microbes.

## Data Availability

The raw data supporting the conclusions of this article will be made available by the authors, without undue reservation.
